# Neglected Disease – African Sleeping Sickness: Recent Synthetic and Modeling Advances

**DOI:** 10.3797/scipharm.1012-08

**Published:** 2011-05-10

**Authors:** Sarvesh K. Paliwal, Ankita Narayan Verma, Shailendra Paliwal

**Affiliations:** 1 Banasthali University, Distt-Tonk, 304022, Rajasthan, India; 2 LLRM medical college, Meerut (U.P.), India

**Keywords:** Human African Trypanosomiasis (HAT), Computer assisted drug design (CADD), Central nervous system (CNS), Structure-activity relationship (SAR), Trypanothione reductase (TR)

## Abstract

Human African Trypanosomiasis (HAT) also called sleeping sickness is caused by subspecies of the parasitic hemoflagellate *Trypanosoma brucei* that mostly occurs in sub-Saharan Africa. The current chemotherapy of the human trypanosomiases relies on only six drugs, five of which have been developed more than 30 years ago, have undesirable toxic side effects and most of them show drug-resistance. Though development of new anti-trypanosomal drugs seems to be a priority area research in this area has lagged far behind. The given review mainly focus upon the recent synthetic and computer based approaches made by various research groups for the development of newer anti-trypanosomal analogues which may have improved efficacy and oral bioavailability than the present ones. The given paper also attempts to investigate the relationship between the various physiochemical parameters and anti-trypanosomal activity that may be helpful in development of potent anti-trypanosomal agents against sleeping sickness.

## Introduction

African sleeping sickness remains one of the most neglected life threatening diseases that have been left untreated till date. Two forms of human African trypanosomiasis (HAT) have been identified that are parasite dependent. The first one *Trypanosoma brucei gambiense (T. b. gambiense)* causes a human chronic infection, endemic in Western and Central Africa while the other *Trypanosoma brucei rhodesiense (T. b. rhodesiense)* has a vast animal reservoir and causes acute illness in people in eastern and Southern African countries. HAT has high occurance in the remote rural areas, where the surveillance is weak or nonexistent, with 50 to 70 thousand estimated cases. According to World Health Organization (WHO) estimation there are half-million cases of HAT or “sleeping sickness” resulting from infections with *T. brucei rhodesiense* and *T. brucei gambiense.* WHO has attributed 50.000 deaths annually to the disease [1]. By more recent estimates, up to 25.000 new cases occur per year, and 50 million people are at risk [2, 3].

Out of six clinically approved drugs for the treatment of HAT, five (suramin, pentamidine, melarsoprol, eflornithine and nifurtimox) have had been discovered more than 30 years ago. Because suramin and pentamidine are ionized at physiological pH, they are unable to cross the blood brain barrier in therapeutic concentrations and are thus used for the treatment of hemolymphatic early stage HAT, caused by *T. b. rhodesiense* and *T. b. gambiense* infections, respectively. The treatment of the second or neurological stage, when the parasites invade the central nervous system (CNS), relies on the organo-arsenical drug melarsoprol and the more recently registered eflornithine. The latter is ineffective against *T. b. rhodesiense* sleeping sickness and is used primarily to control CNS-involved HAT caused by *T. b. gambiense*. All the existing anti-trypanosomal therapies suffer from unacceptable toxicity, poor efficacy, difficulties of administration, and increasing treatment failures due to the development of parasite resistance [4–9]. In the last couple of decades, no new drug has been developed for treatment of early-stage HAT, and only one drug has been developed for late-stage HAT [1, 3, 5]. The need is great for new orally active drugs for the control and eradication of this disease.

Novel medicines are typically developed using a trial-and-error approach, which is time-consuming and costly but yet has the potential to yield new drugs. The application of computer-assisted drug design (CADD) methodologies to this problem has the potential to greatly decrease the time and effort required to discover new medicines or improve current ones in term of their efficacy.

This review focuses on the synthetic and computer-assisted drug design (CADD) approaches made by various research groups for the development of newer anti-trypanosomal agents having improved efficacy and oral bioavailability. The current research also endeavors [10] to investigate the relationship between the various physio-chemical parameters and anti-trypanosomal activity that may be helpful in development of potent anti-trypanosomal agents against sleeping sickness.

## Synthetic advances

Safe, effective and affordable orally active therapies for trypanosomiasis capable of overcoming resistance are required, so the identification of new anti-trypanosomal drug candidates is an urgent priority. Compared to the last 15 years there has been a revival of drug research and development regarding neglected parasitic diseases, and a number of drug development projects are currently ongoing [11]. In view of this, the researchers have endeavored to compile in the present section of the review, diverse series of compounds that have been recently synthesized by various research groups targeting DNA minor groove and many other new targets. Based on the mechanism of action this section has been divided into two sub-sections. The first sub-section includes the series of compounds acting as DNA minor groove binders while in the second sub-section are included all the other anti-trypanosomal compounds and their respective targets. Also are described herein prodrug approaches to provide oral bioavailability for the dication class.

### DNA minor groove binders

**Fig. 1.**
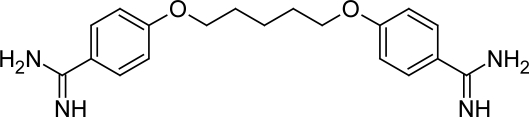
Structure of Pentamidine

The wide range of antifungal and antiparasitic activities related to aromatic diamidines and its excellent results in preclinical and clinical phase of drug development has made aromatic diamidines an interesting class for the development of newer anti-trypanosomal drug therapy. The aromatic diamidines i.e. pentamidine (Figure 1) show their anti-parasitic action by binding strongly to AT-rich sequences in the minor groove of DNA. Therefore DNA minor groove has evolved as a productive target for designing new ant-parasitic drugs. In order to design newer analogues belonging to diamidine series, studies of the DNA complexes with diamidine compounds have been conducted and a number of diamidines have been crystallized with the DNA duplex d(CGCGAATTCGCG)2 which provide valuable models for drug development in the diamidine series. Structures of DNA complexes of furamidine, berenil, and pentamidine, for example, reveal that they all bind in the DNA minor groove at the central AATT sequence. These drugs penetrate deeply into the groove and fit snugly between the walls of the groove. Their amidines form H-bonds with thymine-O2 and/or adenine-N3 acceptor groups on the edges of the bases at the floor of the groove. The amino group of G protrudes into the minor groove and prevents the compounds from assuming their preferred orientation deep in the minor groove. This binding to DNA eventually leads to inhibition of one or more of the several DNA-dependent enzymes (e.g., topoisomerases and nucleases) or direct inhibition of transcription.

For more than 50 years aromatic diamidines and related dicationic molecules have been extensively used but so far only pentamidine [12–15] has been widely employed as a drug in humans despite several adverse effects, such as hypotension, abdominal pain, vertigo, hypersalivation, hypoglycemia, nausea, and mild nephrotoxocity [5–8]. Being a highly flexible molecule that can assume an array of linked conformations related through torsional rotation, changes can be made in the nucleus to produce improved analogous against HAT. In line to this with an aim to increase the efficacy and decrease the side effect related to pentamidine various research groups have made several changes in the pentamidine molecule [16–23] to develop newer compounds having same or enhanced pharmacological activity. The newer analogues of pentamidine that have been recently synthesized are:

#### Pentamidine analogues

**Fig. 2.**
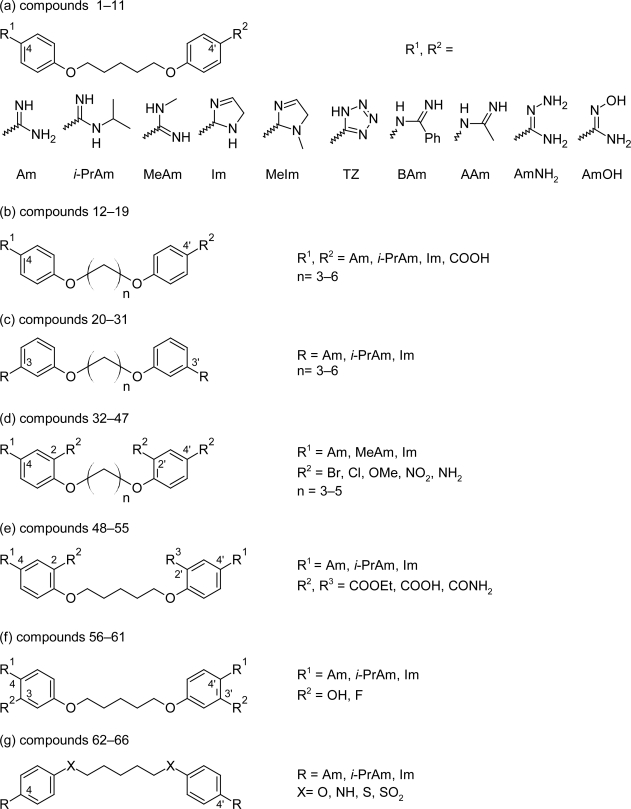
Structures of various congeners of pentamidine synthesized by modification in parent pentamidine molecule

**Fig. 3.**
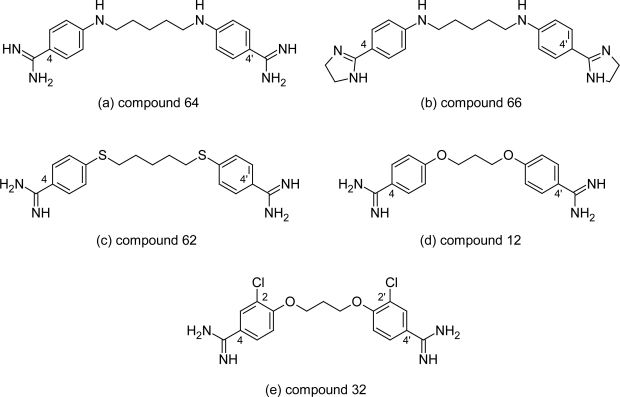
Pentamidine analogues with activity better than that of melarsoprol and/or pentamidine

Large numbers of structurally related congeners of pentamidine were synthesized by Tidwell RR et al [24] by introducing substitutions on the cationic groups, changing the position of dications from 4,4′ to 3,3′ position, changing the length of the aliphatic chain between the two aromatic rings, adding substituent on the aromatic rings at 2,2′ and 3,3′ position and replacing oxygen atoms in the alkyl linker with isosteric sulfur or nitrogen atoms (Figure 2a–g). On comparing the activities of the synthesized compounds with pentamidine and melarsoprol, it was found that few compounds showed activity in the range or better than that of pentamidine and/or melarsoprol. The unsubstituted diamidine compound 64 (Figure 3a) with secondary amino group in place of oxygen atom exhibited subnanomolar activity (<0.001 μM) against *T. b. rhodesiense* and was nearly twice as selective against the pathogen as pentamidine. At the same time the diimidazoline compound 66 (Figure 3b) exhibited the second highest anti-trypanosomal activity in the series with the IC_50_ value of 0.001 μM and also had the highest parasite selectivity (SI_T_= 34500), being 63 times more selective against *T. b. rhodesiense* than pentamidine. The replacement of oxygen atom with sulphur atom as in compound 62 (Figure 3c) also generated active congeners having IC_50_ (0.005 μM) value better than that of pentamidine (0.007 μM). The compounds 20–31 (Figure 2c) in which the cationic groups were present in the 3,3′-position of the aromatic rings and the length of the carbon linker was varied from 3–6 exhibited lower anti-trypanosomal activity compared to pentamidine whereas among the compounds 12–19 (Figure 2b) of the series, in which the amidine groups were present at 4,4′ position and the length of aliphatic chain was varied from three carbon atoms to six carbon atoms, compound 12 (Figure 3d, IC_50_=0.007 μM) with three methylene linker between the aromatic rings had same in vitro activity as that of pentamidine (0.007 μM). Further introduction of 2,2′-dichloro substituent in compound 12 improved the anti-trypanosomal properties as evident from the in vitro activity of compound 32 (Figure 3e, IC_50_=0.004 μM).

These compounds having excellent in vitro activity were evaluated in vivo in the STIB900 mouse model of African trypanosomiasis. The screening was conducted using intraperitoneal dosing at 20 mg/kg daily for four days. The compounds 12, 62 and 64 showed very poor in vivo activity, whereas compound 32 and 66 exhibited excellent in vivo efficacies in the acute mouse model of trypanosomiasis, providing cures of all infected animals.

Thus, because of high selectivity, excellent in vitro and in vivo activity compounds 66 and 32 can serve as a novel lead for further pre-clinical and clinical trials, but its cytotoxcity profile needs to be monitored during its evaluation. Also compound 64 which showed excellent in vitro, in vivo activity and high selectivity index against the *T. b. rhodesiense* merits further SAR optimization in order to synthesized newer lead compounds with reduced cytotoxicity compared to pentamidine.

#### Pyridyl analogues of Pentamidine

**Fig. 4.**
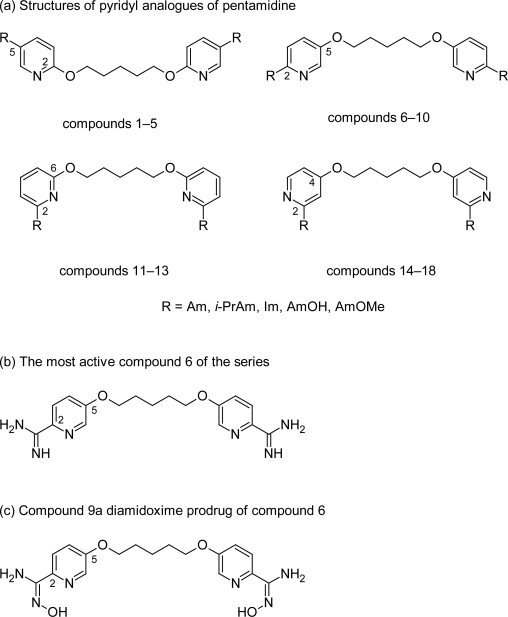
Pyridyl analogues of pentamidine

Increased efficacies of pyridyl analogues of 2,5-bis(4-amidinophenoxy)furan (furamidine) [25–27] encouraged Tidwell RR et al [28] to synthesize pentamidine analogues in which the phenyl rings of pentamidine were replaced with pyridyl fragments. This replacement resulted in series of 18 compounds (Figure 4a), most of which had lower cytotoxicity than pentamidine. SAR study pointed out that the antiprotozoal properties of these compounds depended on the placement of cationic moieties on the pyridine rings as well as the nature of substituents on the amidine groups. The N-substituted congeners were lesser cytotoxic than the unsubstituted diamidines whereas the N-alkylation of cationic fragments reduces the activity of compounds against *T. brucei rhodesiense* compared to pentamidine. A same trend was observed in pentamidine analogues series. The 2, 6-substituted dications (compounds 11–13, figure 4a) and 2,4-substituted dications (compounds 14–18, figure 4a) displayed lower potencies against *T. brucei rhodesiense* than the corresponding 2,5-substituted isomers (compounds 1–10, figure 4a), while among the 2, 5-substituted dications, the compounds possessing cationic substituents adjacent to nitrogen atoms in pyridine rings displayed superior activities against parasites compared to pentamidine as evident from compound 6 (figure 4b) which showed promising anti-trypanosomal activity (0.001μM) and lower cytotoxicity (4.90μM) than pentamidine and melarsoprol, but had poor in vivo activity giving only 1/4 cures in the STIB900 mouse model. However diamidoxime compound 9 (Figure 4c), an oral prodrug of diamidine compound 6 (Figure 4b), exhibited excellent in vivo activity curing four out of four animals upon oral administration in STIB900 mouse model. Although compound 9 did not provide cure in the CNS mouse model of infection but its BBB permeability could potentially be improved by developing prodrug using lipophillic substitutions in place of hydrophilic substitution as used in compound 9. This could provide higher concentration of compound 6 in CNS.

Thus excellent in vitro activity of diamidine 6 and high in vivo efficacy of its prodrug diamidoxime 9 warrant further investigation of these dications as potential anti-trypanosomal drug candidates with improved oral efficacies.

#### Comparison of two most potent pentamidine analogues: compound 6 (Figure 4b) and compound 66 (Figure 3b)

The structure activity relationship of the two series indicates that in both the cases the unsubstituted diamidines were more active than the N-substituted diamidines. The replacement of oxygen atoms in alkyl chain with secondary amino group improves the activity against *T. b. rhodesiense* of pentamidine analogues while in case of pyridyl analogue the compounds with oxygen atoms in the alkyl chain have been the active one. The most potent compound of these two series are compound 6 (Figure 4b) and compound 66 (Figure 3b), which have excellent in vitro activity (0.001μM) better than pentamidine and melarsoprol and also have high selective against the *T. b. rhodesiense* parasite as evident from their selectivity index (SI of compound 6 = 4900 and compound 66 = 34500 μM). Initially it has been believed that the oxygen atom in the aliphatic linker and the amidine groups are important for anti-trypanosomal activity of pentamidine as these groups were part of recognition motif for P2 amino purine transporter in trypanosoma species. Thus the activity of compound 6 can be explained on this basis, but in case of compound 66 the amidine groups have been replaced by imidazoline and the oxygen atom has been replaced by secondary amino groups. Despite these replacements, the compound has shown excellent in vitro and in vivo activity. Thus this is in accordance to recently reported work [29] according to which there is no direct connection between the affinity for P2 carrier and anti-trypanosomal activity.

In view of this, these two compounds can serve as a lead structure for the development of newer analogues of pentamidine with reduced side effect and improved pharmacokinetic profile.

#### Benzofuran derivatives

**Fig. 5.**
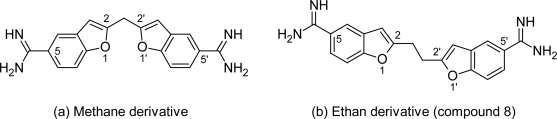
Structure of two bisbenzofuran dications with promising anti-trypanosomal activity

**Fig. 6.**
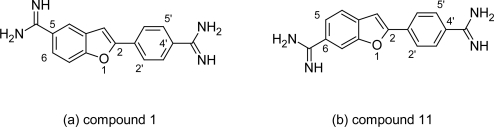
Structure of two phenylbenzofuran dications with promising anti-trypanosomal activity

**Fig. 7.**
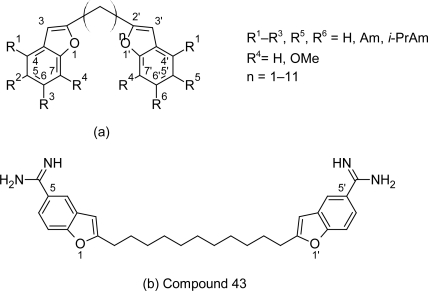
(a) Structure of the basic nucleus of bisbenzofuran and the various substituition used to synthesized 43 congeners. (b) stucture of compound with low cytotoxicity

**Fig. 8.**
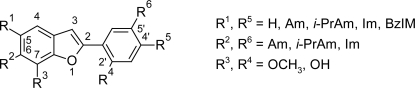
The basic nucleus and the various substitutents used for synthesis of 49 2-phenylbenzofuran cations

In the early 1970s Dann O et al [30–32] reported the anti-trypanosomal properties of two bisbenzofuran diactions (Figure 5a & 5b) and two phenylbenzofuran (Figure 6a & 6b), but since then these compounds received very little attention. However recently lead optimization study of these dications had been carried out by Tidwell RR et al, who have synthesized and reported the in vitro, in vivo activities and cytototixicity of about 43 dicationic bisbenzofuran derivatives (Figure 7a) [33] as heterocyclic pentamidine analogues in which phenoxy fragments were included into a benzofuran structural motif, Tidwell RR et al also synthesized a series of 49 diactionic 2-phenylbenzofuran derivatives (Figure 8) [34].

#### a) Dicationic bisbenzofuran derivatives

Tidwell RR et al found that the in vitro anti-trypanosomal activities of bisbenzofuran derivatives against *Trypanosoma brucei rhodesiense*, and cytotoxicity against mammalian cells depended on the position and the type of cationic substituents as well as the length of the carbon linker between aromatic moieties. As observed in most of the dicationic molecules, the N-substituted congeners were lesser cytotoxic than the unsubstituted diamidines whereas the N-alkylation of cationic fragments reduced the activity of compounds against *T. brucei rhodesiense* compared to pentamidine. At same time the 5-substituted bisbenzofurans were generally less cytotoxic than compounds bearing substituents in the 4- or 6-positions where as the 4-substituted bisbenzofurans were significantly less active against *T. b. rhodesiense* than corresponding 5- and 6-substituted isomers. The in vitro activity of the bisbenzofuran series was not so promising and only lead compound 8, (Figure 5b) exhibited in vitro anti-trypanosomal activity (0.008 μM) comparable to that of pentamidine and melarsoprol. However some compounds from Tidwell series (Figure 7b) showed reduced cytotoxicity profile compared to pentamidine (for example compound 43 has cytotoxicity of 1.85 μM), but had very poor selectivity profile. Thus was proved that the selectivity of bisbenzofurans against *T. b. rhodesiense* decreases as number of methylene groups in the alkyl bridge increases.

However, how the substitution on the amidine groups affects the anti-trypanosomal properties of bisbenzofurans has not been explained. Thus in light of their reduced cytotoxicity compared to pentamidine these molecules require further investigation to study the influence of the type of cationic substituents and the distance between aromatic moieties on uptake and intracellular distribution of dicationic bisbenzofurans in order to understand better the mode of action and thus improve the efficacy of aromatic diamidines.

#### b) Cationic 2-phenylbenzofuran derivatives

**Fig. 9.**
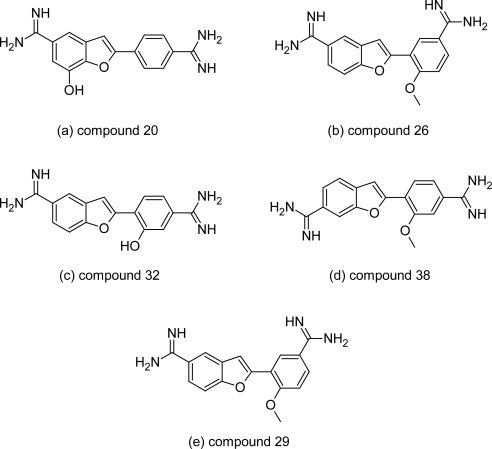
Structures of compounds with promising anti-trypanosomal activity

Tidwell RR et al synthesized and evaluated the activity of 49 dicationic 2-phenyl-benzofurans derivatives (Figure 8) against *Trypanosoma brucei rhodesiense.* Six compounds (Figure 9) among the series including lead compound 1 (Figure 6a) exhibited excellent in vitro and in vivo anti-trypanosomal activity and were highly selective for the *T. b. rhodesiense* parasite. All the six active compounds contained diamidine groups, which clearly suggested that diamidines exhibited higher anti-trypanosomal activities compared to di(*N*-isopropyl)amidines and diimidazolines. The 5,-substituted dicationic compounds 29 (*IC*_50_=0.002 μM, Figure 9e) and 32 (*IC*_50_=0.002 μM, Figure 9c) showed better anti-trypanosomal activity than the 6-substituted dicationic compound 38 (*IC*_50_=0.003 μM, Figure 9d). Whereas among the 5,5′ substituted diamidines and 5,4′ substituted diamidine, selectivity against the parasite of 5,5′-substituted dications was higher than 5,4′-substituted congeners. Introduction of the hydroxy substituents in the 7-or 2′-position of compound 1 (Figure 6a) resulted into compound 20 (Figure 9a) and compound 32 (Figure 9c) with enhanced anti-trypanosomal activity and higher selectivity against *T. b. rhodesiense.* These dicationic compounds were further evaluated in the STIB900 animal model of African trypanosomiasisn. Only compounds 1, 32 and 20 showed exclellent in vivo activity providing 3/4 and 4/4 cures respectively when administered intraperitoneally.

Thus promising in vitro, anti-trypanosomal activity, excellent potency in the acute mouse model of trypanosomiasis and the reduced cytotoxicity (1.9 μM) of compound 1 compared to pentamidine warrants further pre-clinical and clinical trials of this molecule. While compound 32 and 20 can serve as lead compounds for further SAR optimization to derive congener with enhanced activity and lesser cytotoxicity.

#### c) Pentamidine congeners bearing the benzofuran motif

**Fig. 10.**
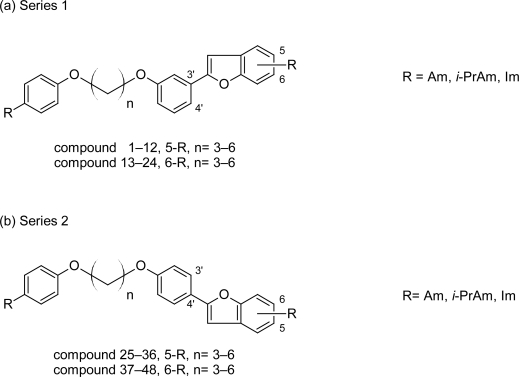
Structures of pentamidine congeners bearing the benzofuran motif

To combine the rigidity of 2-phenylbenzofurans with the flexibility of pentamidine congeners, Tidwell RR et al [35] incorporated the benzofuran ring into molecules of pentamidine-related analogues. A series of 48 pentamidine congeners containing benzofuran fragments (Figure 10a, b) were synthesized and tested in vitro against *T. b. rhodesiense*. Most of the compounds in the series showed cytotoxicity less than that of pentamidine, but had lesser potency and selectivity index as compared to pentamidine. The properties and cytotoxicities of these dications depended on the nature of the cationic substituents, the placement of the benzofuran motif, and the length of the carbon linker. Within the series cytotoxicity of the compounds decreased with the substitution on cationic groups and increased with the elongation of the carbon linker.

Dications with the benzofuran motif in the 4′-position (compounds 25–48, Figure 10b) and connected by the propylene linker are the most potent and more selective against *T. b. rhodesiense* among the series. Thus on the basis of SAR study further improvements can be made in order to produce newer anti-trypanosomal agents with improved pharmacological activity.

#### Comparision of Benzofuran derivatives

The SAR of the three series clearly indicates that the nature of cationic substituents and their position is important in deciding the anti-trypanosomal activity of benzofuran derivatives. The introduction of phenoxy fragment into a benzofuran structural motif may result into retention of affinity for the aminopurine P2 transporter, which could be the reason for anti-trypanospomal activity of benzofuran derivatives. However the precise mechanism by which these compounds show their anti-trypanosomal activity in not known. Among the three series the 2-phenylbenzofuran derivatives are attractive molecules because of their high activity, low cytotoxicity and high selectivity against the HAT parasite. The introduction of phenoxy fragment into the aromatic ring protects the phenylbenzofuran dervatives from metabolic deactivation. This structural modification retains affinity for the aminopurine P2 transporter. The diamidine and the hydroxy or methoxy group may be involved in binding to the DNA minor groove. Thus multiple modes of action may be the reason for promising anti-trypanosomal propertries of 2-phenylbenzofuran derivatives. Thus these compounds require further assessment in order to develop as newer anti-trypanosomal agents with promising pharmacokinetic profile.

#### Furamidine compounds

**Fig. 11.**
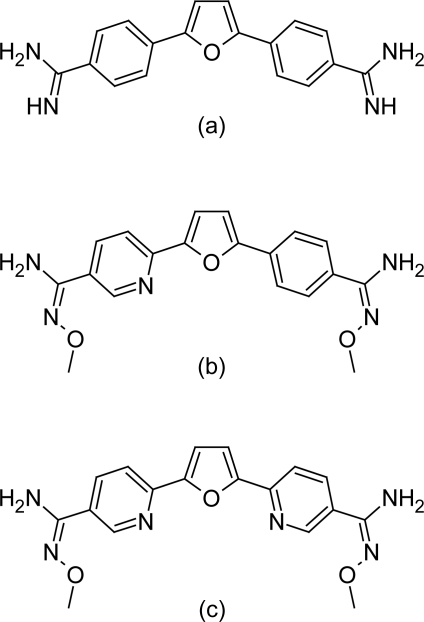
Structure of furamidine and its analogue that have shown promising anti-trypanosomal properties

Since a prodrug of furamidine, 2,5-bis[4-(N-methoxy)amidinophenyl]furan (pafuramidine), has shown promising results in clinical trials against HAT [36], therefore Furamidine (Figure 11a) has gained attention for further evaluation to produce newer anti-trypanosomal analogous. Two other analogues of furamidine (Figure 11b &c) have been evaluated against *T. b. rhodesiense* mouse model and have shown promising results. One approach to enhance the activity of furamidine has been the replacement of the central furan ring with other heterocyclic systems, including thiophene, pyrrole, oxazole, oxadiazole, thiadiazole, pyridazine, methylpyrimidine, and triazine [37–41]. Such structural modification in past has resulted into compound with good anti-parasitic activity. For example in 1977 Boykin DW and Das PB have reported the synthesis and antiprotozoal activity of eighteen substituted 2,5-bis(4-guanylphenyl)furans and related analogues, including “masked” amidines in which the guanyl function was incorporated into a heterocyclic ring. Among these, six compounds have produced cures in mice at submilligram dosage levels and have been somewhat more active in this screen than stilbamidine, hydroxystilbamidine, and pentamidine. In 1994 Boykin DW et al have reported the synthesis of dicationic diaryltriazines nucleic acid binding agents, while in year 1998 Boykin DW et al have also reported the anti-*Pneumocystis carinii* pneumonia activity of dicationic diaryl methylprimidines. Dann O et al in the year 1975 have reported that 3,5-bis(4-amidinophenyl)isoxazole (Figure 13b) which is the most structurally similar to furamidine, is reported to have high activity against a murine *T. brucei rhodesiense* modelc [42]. However most of these compounds have received very little attention since. Recently the optimization of the few of the compounds has been reported and as a result several lead compounds have been synthesized. Here in are listed some of the compounds.

#### a) Dicationic 3,5-diphenylisoxazoles

**Fig. 12.**
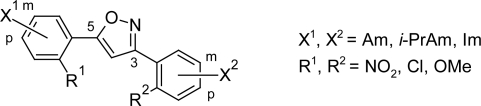
The basic nucleus and the various substitutents used for the synthesis of 43 dicationic 3,5-diphenylisoxazoles

**Fig. 13.**
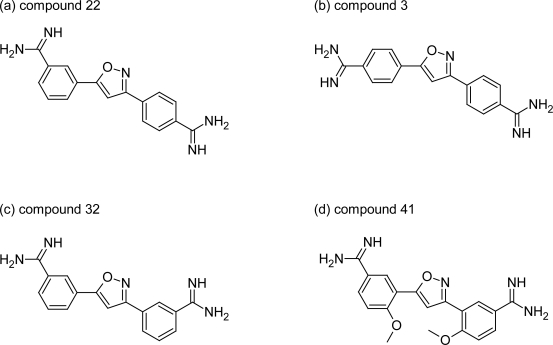
Stuctures of selected dicationic 3,5-diphenylisoxazole derivatives

Tidwell RR et al [43] has recently synthesized a series of 3,5-diphenylisoxazole analogues (Figure 12), in which the central ring of furamidine was replaced by isoxazole, and their activities with furamidine and melarsoprol were compared. Among 43 synthesized dications, the compounds with at least one *p*-amidine moiety (compound 22, Figure 13a and compound 3, Figure 13b), displayed good *IC_50_* values (3.5 nM and 5.1 nM respectively) comparable to that of furamidine (4.3 nM) while loss in potency was observed in compounds with substituted diamidine at *m-*position (compound 32, *IC_50_*=29 nM, Figure 13c). In general, the introduction of nitro, chloro, or methoxy substituents on either aromatic ring resulted in decreased anti-trypanosomal activity. However, the introduction of methoxy group on the aromatic rings of compound 32 resulted into compound 41 (Figure 13b) with comparable anti-trypanosomal activity (4.2 nM) as that of furamidine. Hence these compounds of the isoxazole series that showed good in vitro anti-trypanosomal activity and less cytotoxcity profile relative to furamidine, could be a candidate for further evaluation against animal models of the diseases.

However in vivo evaluation of the present 3,5-diphenylisoxazole series has not been reported yet.

#### b) Cationic 1,4-diphenyl-1H-1,2,3-triazoles

**Fig. 14.**
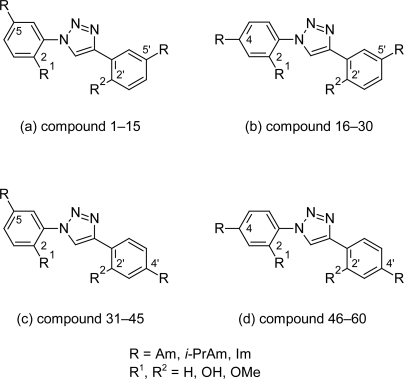
The basic nucleus and position of the various substitution used for synthesis of various analogues of 1,4-diphenyl-1*H*-1,2,3-triazoles series

**Fig. 15.**
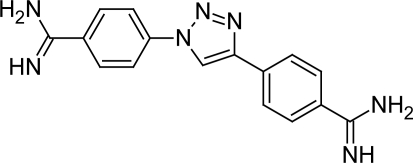
Most active compound 46 of the series with promising anti-trypanosomal properties

The excellent anti-trypanosomal activities of selected cationic diphenylisoxazoles provided the basis of synthesis of a series of 60 cationic 1,4-diphenyl-1*H*-1,2,3-triazoles [44] (Figure 14 a–d) by Tidwell RR et al. The 1,2,3-triazole fragment was used because it was a suitable isosteric replacement for the central five-membered ring of furamidine [45–55] and also because 1,4-diphenyl-1,2,3-triazoles showed geometrical resemblance to furamidine. It was observed that the cytotoxicities of triazoles were as lower compared to that of pentamidine and were not affected by the alkylation on the amidine groups or the substitution on the aromatic rings. However, the placement of the cationic moiety in the 4-position increased the cytotoxicities of diamidines 46 (Figure 15) with respect to the pentamidine. Except few, majority of unsubstituted diamidines exhibited higher in vitro activities against *T. brucei rhodesiense* than bis(*N-*isopropyl)amidines and diimidazolines. The compounds with cationic fragments in the 5,5′-position (compounds 1–15, Figure 14a) of the aromatic rings were in most cases less potent against *T. brucei rhodesiense* than isomers bearing at least one cationic moiety in the 4- or 4′-position (compounds 16–60, Figure 14b–d). However compound 46 (Figure 15) with dicationic moiety at 4,4′ position showed excellent in vitro activity (0.004 μM). The introduction of the hydroxy group in the 2-position of the phenyl ring significantly reduced activities and increased the cytotoxcity against *T. brucei rhodesiense* because of the formation of the intramolecular hydrogen bond between the hydrogen atom of the 2-hydroxy group and the 2-nitrogen of the triazole ring. The in vivo analysis of compound 46 showed that when administered at 4 × 5 mg/kg, 4 × 1 mg/kg and 1 × 10 mg/kg by the intraperitoneal route it cured all the infected animals in the acute mouse model of African trypanosomiasis.

Thus promising in vitro and in vivo anti-trypanosomal activity of cationic 1,4-diphenyl-1*H*-1,2,3-triazoles derivatives and in particular compound 46 necessitate further investigation of this class of compounds as potential anti-trypanosomal drug candidates.

#### Comparision of isoxazole and triazole derivative of furamidine

The replacement of central furan ring of furamidine either by isoxazole or 1,2,3-triazole resulted into compounds with better activity than that of furamidine. Position and the nature of the cationic substituent governed the anti-trypanosomal activity of the two series. In general the para substituted diamidines in both the series were the most promising compounds with good activity. Thus these compounds should be further evaluated to produce better analogue of furamidine

#### Bis(2-aminoimidazoline) and Bisguanidine

**Fig. 16.**
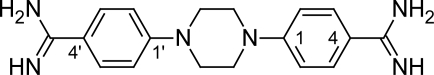
*N*,*N*′-bis(4-amidinophenyl)piperazine

**Fig. 17.**
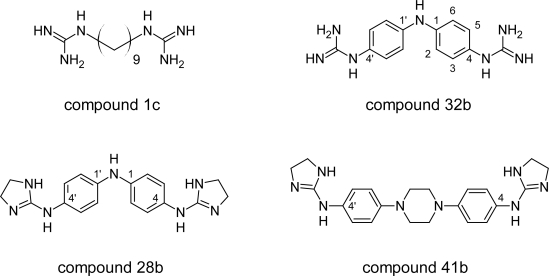
Structures of compounds that showed excellent in vitro activity and selectivity

The recent report on *N,N'*-bis(4-amidinophenyl)piperazine (Figure 16), which was shown to be very effective in vivo anti-trypanosomal agent has attracted the interest in this compound [56]. Hence in search for new HAT chemotherapy Dardonville C et al [57] decided to carry out an in vitro screening of a total of 62 compounds against the parasite *T. brucei rhodesiense* taken from their in-house library. Based on this they showed that bisguanidine and especially bis(2-aminoimidazoline)diphenyl compounds displayed potent anti-trypanosomal activity in vitro and vivo against *T. b. rhodesiense*, the causative agent of acute HAT [58, 59]. Among the 62 compounds screened, compounds1c, 28b, 32b and 41b (Figure 17) showed excellent in vitro activity (49nM, 69 nM, 22 nM and 118 nM respectively) as well as high selectivity (>5294, 3072, 29.5 and 881 respectively) for the parasite. These studies revealed that compounds bearing 2-aminoimidazoline cations had higher selectivity for the parasite and similar activities with respect to their guanidine counterparts. In addition, a correlation between anti-trypanosomal activity and DNA binding affinity was observed, suggesting a possible mechanism of action for these compounds. Those molecules that showed an excellent in vitro activity as well as high selectivity for the parasite represent new anti-trypanosomal lead compounds.

In light of these promising results, bis(2-aminoimidazoline) derivatives deserve more investigation as anti-trypanosomal agents and DNA minor groove binders. The synthesis and study of new derivatives and prodrugs of these lead compounds is ongoing.

### Other target and lead compounds

Progress has also been made on targets other than DNA minor grove. In addition to newer targets some new lead compounds have also been identified with proper anti-trypanosomal activity but uncertain mechanism of action.

#### Trypanothione Reductase Inhibitors

The enzyme trypanothione reductase (TR) which restores the oxidized trypanothione to reduced state has evolved as an effective drug target. Many inhibitors of this enzyme have been developed but most of them combat with problems related to bioavailability, pharmacokinetics and metabolism. Despite this quinolines have evolved as most important class of compounds against TR due to their broad spectrum of activity, excellent pharmacological and pharmacokinetic properties such as high plasma levels, high clearance, oral and parenteral applicability, chemical stability and rare side effect.

**Fig. 18.**
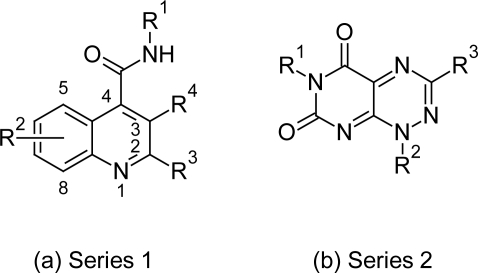
Two novel series active against the enzyme trypanothione reductase

**Fig. 19.**
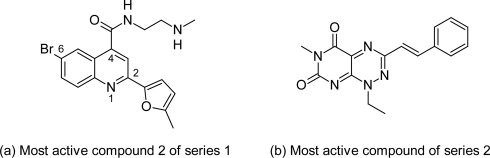
Most active compounds of the two series

In view of this Gilbert IH [60] reported the screening of 62000 compound libraries against *T. brucei*. High through put screening (HTS) which resulted into identification of two novel compound series active against the enzyme trypanothione reductase. Series 1 was based on the quinoline scaffold (Figure 18a) in which compound 2 (Figure 19a) with methylfuran group at R^3^ position, Br at 6^th^ position of quinoline ring and *N*-methylethanamine group at R^1^ position was the most potent and significant TR inhibitor with TR inhibitory activity of 1.1 μM. According to SAR study the replacement of methylfuran with other groups like furan, phenyl, pyridinyl, thiophene led to decrease in activity. Also the replacement of Br with H or F reduced the activity while Cl group retained the activity. At 4^th^ position the NH and NMe groups were equally active. The alkylamines at R^1^ were active whereas the simple alkyl or aryl groups led to inactive compounds. The second series was based on the pyrimidopyridazine scaffold (Figure 18b), in which the compound 49 (Figure 19b) showed promising inhibitory activity of 2.6 μM. In case of series 2 replacement of methyl group at R^1^ position by H led to the decrease in activity. At R^2^ position methyl and ethyl group showed activity while the H and chain extensions to propyl, butyl and cyclopentane led to decrease in activity. Substituted phenyl and alkenylphenyl group at R^3^ position gave the most active compound of the series. Thus these quinoline compounds with promising activity could serve as lead compounds for further development of targeted drugs against African trypanosomiasis.

**Fig. 20.**
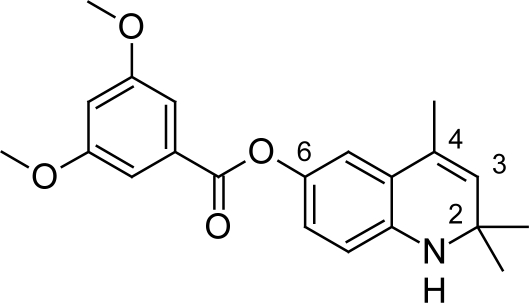
Lead anti-trypanosomal compound 1,2-dihydro-2,2,4-trimethiquinolin-6-yl 3,5-di-methoxybenzoate

**Fig. 21.**
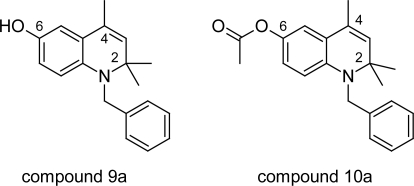
Compound 9a and its prodrug 10a having promising anti-trypanosomal properties

In addition to this synthetic optimization study based on the lead anti-trypanosomal compound 1,2-dihydro-2,2,4-trimethylquinolin-6-yl 3,5-dimethoxybenzoate (Figure 20) was undertaken by Werbovetz KA et al [61] in an attempt to discover new trypanocides with potent in vivo activity targeting TR enzyme. In course of this a total of 53 compounds were evaluated in vitro for their anti-trypanosomal activity and cytotoxicity. The compounds with oxygen atom at 6^th^ position were the most active compounds in the series, for example compound 9 (Figure 21) showed better anti-trypanosomal activity (0.007 μM) and low cytotoxicity (6.8 μM) than melarsoprol (*IC_50_* = 0.008μM and cytotoxicity = 7.9μM). Whereas compounds lacking the 6-oxygen atom or bearing an oxygen atom at the 7-position rather than the 6- position were far less potent than those containing an alcohol or acyloxy group at the 6-position. Compounds carrying aliphatic or a 2-phenylacetyl ester side chains were as potent and selective as their benzoylated or acetylated counterparts. However compound 9a was unstable due to auto-oxidation, so the unstable alcohols were esterified to generate prodrug 10a (Figure 21) having promising activity of 0.014μM against these parasites and a selectivity index of 1700. The in vivo evaluation of compound 10a in a murine model of African trypanosomiasis showed good results as the prodrug extended the lifespan of mice infected with *T. b. brucei*.

Thus compound 10a can serve as lead compound for further investigation of this class of molecules as potential candidates against HAT. Efforts are also be undertaken to further elucidate the metabolism, pharmacokinetics, and the anti-trypanosomal mechanism of action of this novel and promising class of compounds.

#### Topoisomerase inhibitors

DNA topoisomerases have evolved as an effective drug target in prokaryotic and eukaryotic systems as these enzyme mediate mechanistic interactions such as supercoiling, relaxation, knotting or catenating of DNA double helices. Based on their mechanism of action, topoisomerases can be classified as type I enzymes, which break a single strand of the DNA helix during the catalytic cycle, and type II enzymes, which make double-stranded breaks. On the basis of primary sequence and reaction mechanism, type I topoisomerases are further subdivided into type IA and type IB.

**Fig. 22.**
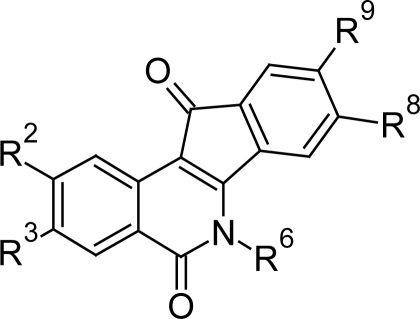
The indenoisoquinoline scaffold. Structure-activity analysis was performed with substitutions at the numbered positions

**Fig. 23.**
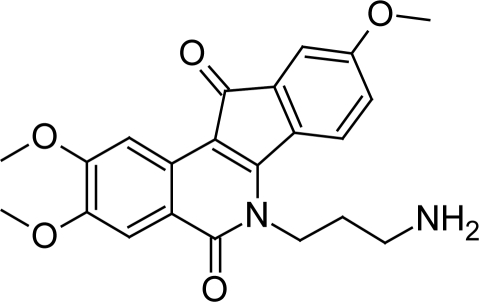
Lead compound 12 with promising in vitro and in vivo activity

Recently Shapiro TA et al [62] evaluated the activity of indenoisoquinolines (Figure 22), originally known to have anti-cancer activity, against *T. brucei* and found that most of the compounds showed in vitro activity at submicromolar concentrations. The compound 12 (Figure 23) with propylamino group at R^6^ position and methoxy group at R^2^, R^3^ and R^9^ was the most active one with in vitro anti-trypanosomal activity of 0.05 μM. The compound also showed good in vivo activity as it delayed parasitemia and extended survival in infected mice. According to structure-activity analysis the compounds with enhanced potency included alkylamino substitutions on N-6, methoxy groups on C-2 and C-3, and a methylenedioxy bridge between C-8 and C-9. Testing of indenoisoquinolines with promising activity on L1210 mouse leukemia cells revealed all the compounds were more effective against trypanosomes than against mammalian cells. The indenoisoquinolines also showed appreciable water solubility indicating that these compounds have good quality for drug development. These compounds showed their anti-trypanosomal action by multiple mechanisms. The study indicated that they stabilize topoisomerase-DNA complexes in situ and may also impede topoisomerase binding to DNA. These agents markedly inhibited DNA synthesis by interfering with topoisomerase and possibly other DNA-metabolizing enzymes.

Thus multiple mechanisms of action of these compounds against trypanosomes are an attractive feature, since such compounds are less prone to drug resistance. Therefore indenoisoquinoline compound 12 is a promising lead for the development of drugs against sleeping sickness.

**Fig. 24.**
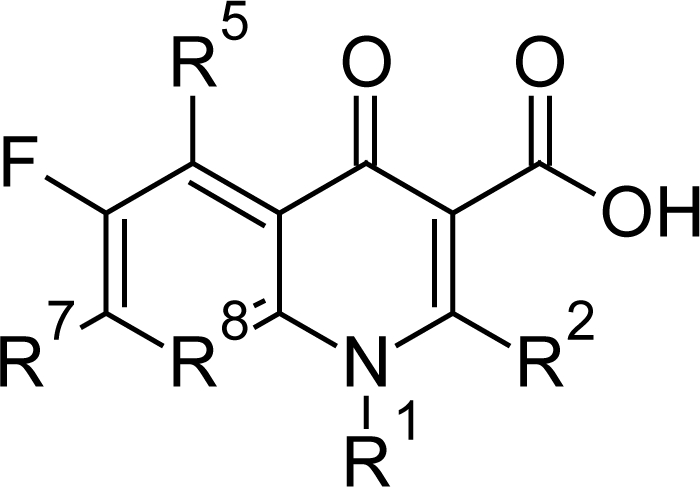
Basic chemical structure of fluoroquinolones

**Fig. 25.**
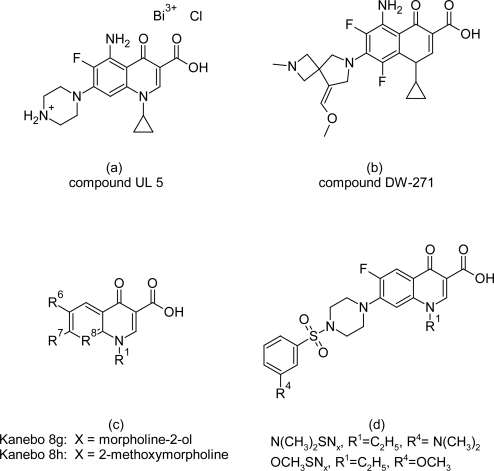
Most promising compounds of quinolone derivatives

In addition Burri C et al [63] tested about 160 fluroquinolones and their derivatives which are specific inhibitors of topoisomerases, against cultured bloodstream forms of *T. b. rhodesiense*. The modifications in the quinolone core nucleus (Figure 24) led to characteristic in vitro activity and toxicity. The metal quinolone UL 5 (Figure 25a) with F group at the position 6 and piperazin at R^7^ position was the most potent compound with *IC_50_* values of 100 ng/ml. DW 271 (Figure 25b), thiazolopyrazine incorporated tetracyclic quinolines Kanebo 8g and Kanebo 8h (Figure 25c), and the two sulphanyl fluoroquinolones N (CH_3_)_2_SNx and OCH_3_SNx (Figure 25d) were also active, with *IC_50_* concentrations in the range of 300 ng/ml up to 900 ng/ml. Some of the DuPont compounds, developed as anti-tumour drugs, were highly active but also showed high cytotoxicity on HT-29 cells. It was observed that the position R^1^ and position R^2^ in the quinolone core nucleus was not prerequisite for anti-trypanosomal activity and also substitution at R^8^ position was not necessary for trypanocidal activity. Thus, based on the result obtained from SAR analysis special attention should be given to R^7^ position and the tetracyclic derivatives. The in vivo results of these compounds were very poor, as none of the compounds evaluated produced cure of mice in dose escalation experiment up to 100mg/kg i.p. However no signs of toxicity were observed during the experiments. The in vivo ineffectiveness had not been explained and no drug level determination in the plasma of the treated mice was performed.

Despite these failures, this class of compounds is very attractive for further search for trypanocidal compounds because of complete lack of any toxic signs at all dosage levels tested in vivo.

#### Polyamine metabolism inhibitors

Polyamines are generally involved in growth and differentiation [64–67] within the cell and their analogs are also used as anticancer agents, antiparasitic agents, antidiarrhoeals, anti-HIV agents, metal chelators, and gene delivery agents. Since the inhibition of the initial polyamine biosynthesis enzyme, ornithine decarboxylase, by DL-α-difluoromethyl-ornithine (DFMO) is toxic to African trypanosomes cells, [68, 69] polyamines can become a promising anti-trypanosomal compound.

DFMO [70–72] is the most recently developed agent for late stage *T. b. gambiense* and *T. b. brucei* sleeping sickness, but has not been active against all strains of *T. b. rhodesiense*. The major drawbacks of DFMO are its cost, the duration of treatment and its availability. Recent clinical studies have investigated that DMFO can be used in combination with clinically used trypanocides including suramin, nifurtimox and melarsoprol [73, 74]. These combinations result in significant reduction in DFMO dosage and time of administration. Initial clinical study has shown that DFMO + nifurtimox are superior to DFMO + melarsoprol and melarsoprol + nifurtimox. The DFMO + nifurtimox regimen (NECT regimen) allowed reduction in DFMO regimen from 14 to 7 days (56 versus 28 infusions) with a 94% cure rate and are associated with significantly reduced adverse side effects as compared to melarsoprol-based therapy. The success of the combined regimen has been most likely due to ability of DMFO to reduce trypanothione levels and resistance to oxidative stress and the ability of nifurtimox to generate oxidative stress.

In addition to DFMO, a number of other agents targeting polyamine metabolism have shown promise. These include MDL 73811 (5′-{[(2*Z*)-4-aminobut-2-en-1-yl](methyl)amino}-5′-deoxyadenosine), an enzyme-activated inhibitor of S-adenosylmethionine (AdoMet) decarboxylase which supplies decarboxylated AdoMet, the source of aminopropyl groups for spermidine and spermine synthesis [75].

**Fig. 26.**
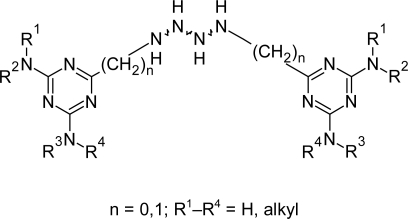
Lead structure A

**Fig. 27.**
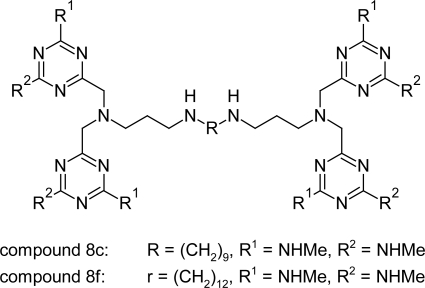
Tetra triazine substituted polyamine compounds

Recently Gilbert et al [76] designed, synthesized, and evaluated substituted polyamines, carrying 1,3,5-triazine units, as potential anti-trypanosomal drugs. Preliminary results indicated that this route might be successful, and lead structure A (Figure 26) was used as a starting point for the synthesis of two series of analogues. In the first series, the influence of structural changes of the central core unit was investigated while in the second series, the effect of additional methyl substituents on the 1,3,5-triazine was studied. The compounds were designed with the intention to selectively target the interior of *T. brucei* via the P2 amino-purine transporter. In the first series the compound containing the *n*-dodecyl chain as core unit, showed weak activity against *T. b. rhodesiense*. The compound with *n-*nonyl chain was the most promising compound and its various analogues were designed by replacing NH_2_ groups on the triazine ring with NHMe and NMe_2_ groups. Introduction of one or two methyl groups per triazine unit resulted in a 10-fold increase in anti-trypanosomal activity. When four methyl groups per triazine unit were introduced, an 80-fold increase in activity was observed. Similarly, replacement of NH_2_ group in *n-*dodecyl chain led to 2–20-fold higher anti-trypanosomal activity for the methylated derivatives. Monosubstituted compounds showed a slight increase in activity against *T. b. rhodesiense* as compared to the disubstituted compounds. The methylamino substituted triazines attached to the C9- (compound 8c, Figure 27) or C12-(compound 8f, Figure 27) polyamine precursor via an additional CH_2_ linker resulted in most active trypanocidal compounds(*IC*_50_ of 8c = 0.27μM and 8f = 0.18 μM). Beside good activity, the compounds showed poor in vivo activity producing no cure to the infected mice and concentrations greater than 10 mg kg^−1^ induced severe acute toxicity.

The actual mode of action for the reported triazine substituted polyamines remains unclear. So to understand and improve the activities of these compounds, further research has to verify intracellular drug targets and possible metabolic pathways.

#### AdoHcy hydrolase inhibitors

Stanislaw FW et al [77] have reported the anti-trypanosomal activity of 5′-deoxy-5′-(*E*)-(iodomethylene)adenosine, which is a known inhibitor of AdoHcy hydrolase, [78, 79] and the synthesis and inhibition of *T. brucei* by a series of 6-*N*-cyclopropyladenosine analogues modified at carbon-5′ including halomethylene, acetylenic, and oxime derivatives.

**Fig. 28.**
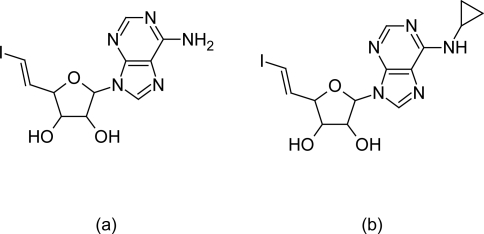
(a) structure of 5′-deoxy-5′-(*E*)-(iodomethylene)adenosine (b) structure of 6-*N*-cyclopropyl-5′-deoxy-5′-(*E*)-(iodomethylene)adenosine

The 5′-deoxy-5′-(*E*)-(iodomethylene)adenosine (EIDDHA) and its 6-*N* cyclopropyl analogue (Figure 28a, b) have shown promising in vitro inhibitory activity (IC50 at 9 and 12 μg/mL) against *T. brucei*. The utilization of adenosine analogues as anti-parasitics should be explored as a therapeutic paradigm, as it has been shown previously that inhibitors of AdoHcy hydrolase are also very potent inhibitors against the growth of *Plasmodium falciparum* [79]. This class of 6-*N*-cyclopropyl adenosine analogues modified at carbon 5′, does not exhibit an inhibitory effect on human or parasite forms of the enzyme and displays only marginal antiviral activity in comparison to analogues which have been unmodified at 6-amino position (that are potent inhibitor of AdoHcy hydrolase). Therefore these compounds require further structural modification in order to develop newer analogues with improved activity against *T. brucei*.

#### New 4-[3-(4-phenoxyphenyl)-1H-pyrazol-5-yl]morpholine derivatives

**Fig. 29.**
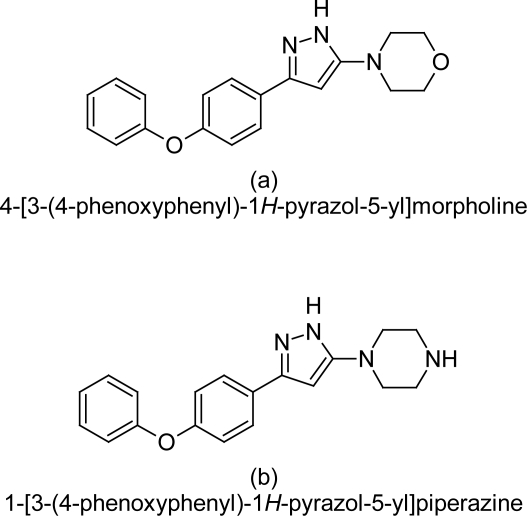
The most active compound of morpholine series

The synthesis of 4-[5-(4-phenoxyphenyl)-2*H*-pyrazol-3-yl] morpholine derivatives by Perozzo R et al [80] resulted in to the discovery of newer class of anti-trypanosomal compounds having stage specific action, as these compounds have shown moderate to very good activity against the blood stage of *T. b. rhodesiense.* The two compounds, 4-[3-(4-phenoxyphenyl)-1*H*-pyrazol-5-yl]morpholine (1.0μM) (Figure 29a) and 1-[3-(4-phenoxyphenyl)-1*H*-pyrazol-5-yl]piperazine (1.1 μM) (Figure 29b) with a pyrazol ring, are the most potent anti-trypanosomals of the series and have same cytotoxicity (61.6 μM), indicating that the pyrazol ring is very important for anti-trypanosomal activity. The substitution of pyrazol ring with isoxazole derivative leads to a six fold reduction in activity as compared to the most potent compound. In addition, substitution with nitrophenyl or aminophenyl also results in strong reduction in activity. The phenoxy ring in the compounds is also important for activity as replacing it by an ethylene group results in nine fold reduction in efficacy. Further substitution with a nitro group or an amino group reduces potency up to 4-fold or 18-fold respectively. The stage specific action of these compounds is unknown. Further optimization of this class of compounds is required in order to lower the cytotoxicity profile of the compound.

#### Glutathione derivatives

**Fig. 30.**
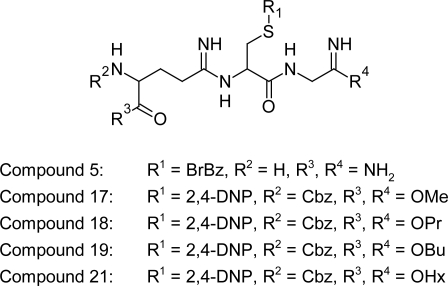
N,S-blocked glutathione diesters

In vitro evaluation of a series of N-, S-, and COOH-blocked glutathione derivatives have been carried out by D'Silva and Daunes [81] against bloodstream form *Trypanosoma brucei* trypomastigotes, to identify the determinants necessary for activity and for further development into an active lead structure. The results shows that N, S-blocked glutathione diesters are the most active inhibitors of *T. brucei* parasites and that *N*-acetyl-*S*-benzyloxy-carbonylglutathione dimethyl ester (compound 5) and the *N,S*-benzyloxycarbonyl-*S*-2,4-dinitrophenylglutathione diester derivatives (compounds 17–19 & 21) (Figure 30) represent lead structures possessing minimal toxicity which potentially could be developed further to yield a therapeutically active agent for the treatment of trypanosomiasis.

### Prodrug approaches

Pentamidine, furamidine and its analogues lack oral bioavailability [82, 83].In addition several analogues of furamidine show excellent activity on intravenous dosing but are ineffective on oral administration [83, 84]. Generally, oral administration is the preferred dosing regime, and hence, prodrug strategies for diamidines that have the potential to overcome their limited oral bioavailability merit attention. The following works have been performed by Boykin DW et al to develop prodrugs.

#### O-Alkoxyamidine Prodrugs of Furamidine

Boykin DW et al [85] syhthesized and evaluated five O-alkoxyamidine analogues of the prodrug 2,5-bis[4-methoxyamidinophenyl]furan against *Trypanosoma brucei rhodesiense* in the STIB900 mouse model by oral administration. It was observed that the size of the O-alkyl side-chain determined the metabolic stability of the prodrugs. The prodrugs with the O-methyl analogue were most susceptible to metabolism while the larger O-*n*-butyl and *O*-*n*-hexyl groups were least susceptible to metabolism. The in vivo studies in the STIB900 mouse model for *T. b. rhodesiense* showed that compounds with an O-methoxyamidine or O-ethoxyamidine group effectively cured all trypanosome-infected mice, whereas prodrugs with larger side-chains did not completely cure the mice. Therefore the *O*-alkoxyamidine prodrugs, where the alkyl chain is less than three carbons, could effectively be used as prodrugs for amidines.

#### Aza-Analogues of Furamidine

**Fig. 31.**
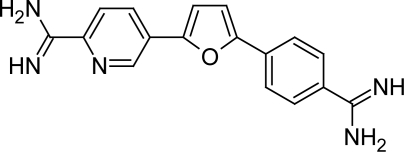
Compound 8c

Boykin DW et al [26] have also prepared aza-analogues of furamidine that exhibit high in vitro activity against *T. b. rhodesiense.* Several prodrugs of these aza-analogues (8c) (Figure 31) show excellent oral activity in vivo which is superior to that of furamidine, 2,5-Bis[4-(methoxyamidino)phenyl]furan and 2,5-bis[4-(*N*-hydroxyamidinophenyl]furan against *T. b. rhodesiense* in this mouse model. They have found several excellent candidates for further evaluation against *T. b. rhodesiense.*

#### Terphenyl Diamidines and Analogues

**Fig. 32.**
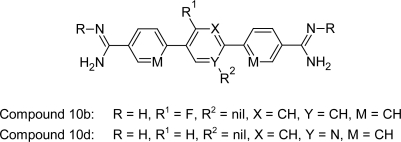
Terphenyl diamidines and analogues

In addition to above mentioned prodrug synthesis Boykin DW et al [86] also reported that bis-amidoximes and bis-*O*-alkylamidoximes of a number of diamidine systems are effective prodrugs. In order to develop orally effective anti-trypanosomal agents, they synthesized these two types of potential prodrugs in the terphenyl series. It was found that compound 10b and 10d (Figure 32) showed good activity in the range of 2 nM. Among these compounds 10b had lower cytotoxicity (6.4μM) profile but had very poor in vivo activity (cured none of the infected animal in STIB900 mouse model). Whereas compound 10d showed excellent in vivo activity, by curing all the infected animals upon oral administration in STIB900 mouse model. To capitalize on the efficacy of these potent dications, other prodrugs that rely on different bioconversion pathways need to be developed.

#### Fused ring dicationic compounds and their prodrugs

**Fig. 33.**
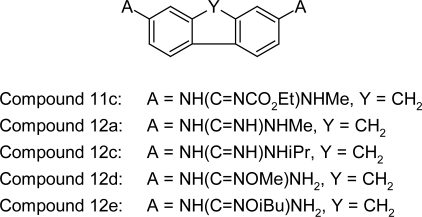
Fused ring dicationic compounds and their prodrugs

Boykin DW et al [87] showed that some of the dicationic guanidine, *N*-alkylguanidine, and reversed amidine derivatives of fused ring systems have good in vitro activity against *Trypanosoma brucei rhodesiense* [87, 88]. The dicationic *N*-isopropylguanidino-9*H*-fluorene (12c, Figure 33) showed promising in vivo biological results by giving 4/4 cures of the treated animals in the STIB900 animal model for African trypanosomiasis. In addition the *N*-methyl analogue (12a, Figure 33) also showed high activity giving 3/4 cures of the treated animals in the STIB900 animal model for African trypanosomiasis. In order to enhance the oral bioavailability, two novel classes of potential guanidine prodrugs were prepared. The *N*-alkoxyguanidine derivatives 12d and 12e (Figure 33) were not effective as prodrugs. Whereas the carbamate prodrugs (11c, Figure 33), gave promising results with 4/4 cures on oral administration in the STIB900 mouse model. The result showed that these compounds bind strongly to the DNA minor groove, but despite strong bonding these compounds do not have high antiparasitic activity.

## Computer-assisted drug design (CADD) approaches

As compared to the last 15 years, there has been a revival of drug research and development regarding neglected parasitic diseases, and a number of drug development projects are currently ongoing. However discovering lead compounds with anti-trypanosomal activity remains a crucial step to sustain the progress achieved till date. The use of Computer-assisted drug design (CADD), since their start, has become increasingly helpful in understanding many aspects of chemical-biological interactions in drug and other scientific research. The latest technological advances (QSAR, structure-based design, ligand-based design, cheminformatics & bioinformatics) are providing a much improved basis for the design of ligands and inhibitors with desired specificity. Recently, computer-assisted drug design approaches based on ligand-based and structure- based drug design have been successfully employed to develop new drugs for the treatment of cancer, AIDS and other diseases [89–96]. QSAR [97] has been widely used for years to provide quantitative analysis of structure and activity relationships of compounds. Statistical methods are applied in QSAR modeling to establish correlations between chemical structures and their biological activities. Once validated, the findings can be used to predict activities of untested compounds. Though only countable but sustainable attempts has been made to build molecular model in the field of HAT, exemplified by the work of Prashanth A et al [98] who have performed 3D QSAR analysis on a library of heterocyclic diamidines derivatives with antiparasitic activity and Martin MB et at [99] who have performed CoMFA analysis on bisphosphonates against *Trypanosoma brucei rhodesiense.*

Prashanth A et al have performed CoMFA and CoMSIA based 3D QSAR analysis on furamidine and a set of 25 other structurally related compounds. The main goal of this study has been to correlate the structural features of this class of compounds with their biological activity using the knowledge of the DNA minor groove bioreceptor in AT sequences.

Two different alignment strategies, based on a putative kinetoplast DNA minor groove target, have been used. Due to conserved electrostatic properties across the compounds, models that used only steric and electronic properties have not performed well in predicting biological results. An extended CoMSIA model with additional descriptors for hydrophobic, donor, and acceptor properties have had good predictive ability with a *q*^2^ = 0.699, *r*^2^ = 0.974, SEE, standard error of estimate = 0.1, and *F* = 120.04. The results have been used as a guide to design compounds that, potentially, have better activity against African trypanosomes.

These specifically derived chemical properties that are important to activity and in view of this, the developed model can be adopted as a rational approach toward the selection of substituents at various positions in the scaffold. The information can be used to predict new compounds for synthesis that have high probability of enhanced activity.

Martin MB et at have been the first to provide evidence that the enzyme farnesyl pyrophosphate synthase is a principal site of action of the bisphosphonate risedronate in inhibiting the growth of bloodstream-form *T. b. rhodesiense* trypomastigotes, the causative agent of human East African trypanosomiasis. They have also reported 3D-QSAR/CoMFA analysis of 26 bisphosphonates, yielding a theoretical-versus-experimental pIC50 correlation of *R*^2^ = 0.87 with a cross-validated *R*^2^ value of 0.79. This has been the first report of a QSAR/CoMFA correlation for 1,1-bisphosphonates acting as antiparasitic agents. These results should be of use in the design and further development of bisphosphonates as antiparasitic agents.

Daunes and D'Silva [100] have performed QSAR analysis in order to derive the contribution of Log P and Es to the in vitro antiprotozoal activity of glutathione derivatives. Analysis of the inhibition data (*ED_50_*) vs calculated log *P* and *E*s values provided evidence to support membrane penetration and steric factors as the key component in the activity of these compounds. The optimum values for log *P* and *E*s determined have been 5.8 and −0.70, respectively. The best linear QSAR equation derived by a process of elimination, using a set of 17 compounds, excluding the outliers is eq 1 with an *R*^2^= 0.84 and *F=* 12.0

(1) $${\text{Log}(1/\text{ED}}_{50})\;=\;-2.57\;\text{log}P\;+\;1.87\text{Es}+0.20\;\text{MW}-\;0.002\;W\;-\;102\;$$

Equation 1 indicates that the therapeutic activity of these compounds decreases with increasing values of log *P* and *E*s.

Our core research group has also performed QSAR analysis of dicationic diphenylisoxazoles [10]. In this study, attempt has been to investigate the relationship between the various physiochemical parameters and anti-trypanosomal activity of dicationic 3,5-diphenylisoxazoles that may be helpful in development of potent anti-trypanosomal agents against sleeping sickness.

Several statistical expressions have been developed using stepwise multiple linear regression analysis (MLR) and partial least squares (PLS). The best MLR model showed good correlative and predictive ability as shown in following equation

$$\text{Y }=\;-0.023566637\;X_{1}\;-\;0.32909143\;X_{2}\;-\;0.090104289\;X_{3}\;-\;1.0970852$$

Where X_1_=cosmic energy, X_2_=Log P, X_3_=total lipole.

s value=0.31, F value=79.01, r=0.94, r^2^=0.88, r^2^cv=0.84

A comparable PLS model with r^2^_cv_=0.84 has also been obtained. The developed model has been validated by leave-one-out method of cross-validation and prediction of test set. The study indicates that the anti-trypanosomal activity is largely explained by cosmic energy, log P and total lipole descriptors. The QSAR study has reported in present study provides important structural insights, related to anti-trypanosomal activity. Authors have developed a validated and highly predictive model sharing important structural requirement for effective binding of anti-trypanosomal compounds to minor groove of *T. b. rhodesiense* DNA. The model reported in the study should be helpful in development of new compounds with improved efficacy and oral bioavailability. In line to the developed model authors have also designed some molecules which showed good activity in silico. The further study of these compounds is in progress.

## Conclusion

The poor pharmacokinetic profile and toxicity produced by the drugs currently used to treat trypanosomiasis requires an immediate attention for the development of safe, economical and high affinity chemotherapeutic agents to meet the need for this class of drugs. The unacceptable truth is the lack of attention of the government and less interest of pharmaceutical companies in light of less monitory gain in the area of protozoal infection since most of the affected people belong to poorer country. Surprisingly the endeavors of different research group discussed in this review belong to academic institutions. It is felt by the researchers that the treatment for HAT requires a combined approach by government organization, pharmaceutical companies and academic institutions. The most promising fact is that in the last decade several synthetic approaches have been made in the field of development of anti-trypanosomal therapies. Several compounds are being synthesized that yield new compounds for the treatment of HAT, as a result of these synthetic approaches newer leads have been identified, which are under different phase of drug development.

The promising in vitro and in vivo activities of dicationic molecules clearly indicate that aromatic diamidines are the most promising class of compounds for the development of newer drugs against HAT. The most important one is the newer analogous of existing drugs pentamidine (compounds 32, Figure 3e and 66, Figure 3b) which shows excellent in vivo and in vitro activity (0.004 and 0.001 μM respectively) and also has very good selectivity index against the parasite but is cytotoxic. In an attempt to synthesise newer compounds with reduced cytotoxicity than pentamidine molecules, several benzofuran derivatives have been synthesized. However the replacement of phenoxy fragement of pentamidine with a benzofuran motif has resulted in poor analogues with lower anti-trypanosomal activity but improved cytotoxic profile. However the strong activity against *T. b. rhodesiense* isolates indicates that steps should be taken to initiate further studies of compound 66 and compound 32 which can be further evolved as new lead compound. The newer analogues of furamidine also show promising anti-trypanosomal activity along with lower cytotoxicity than furamidine. Thus strong activity against *T. b. rhodesiense* and lower cytotoxicity of compound 3 (Figure 13b) indicates that these compounds should be further evaluated. Thus the DNA minor grove binders are still the most interesting and potential target for the development of newer anti-trypanosomal agents.

Apart from diamidines, polyamines and guanidine also have the potential to give anti-trypanosomal compounds. The success of DMFO as polyamine inhibitors has attracted researchers for synthesis and development of agents targeting polyamine metabolism. Recently polyamines carrying 1,3,5-triazine units have been synthesized and evaluated against *T. brucei*. These compound exhibited good activity but are cytotoxic. Morpholine and dihydroquinolines have also shown promising in vivo anti-trypanosomal activity. In addition to this development of prodrugs, the existing compounds also promise to address the pharmacokinetic related problems, in near future.

Thus excellent in vitro and in vivo activities and high selectivity of aforementioned compounds merit further investigation in order to reduce the cytotoxicity that may result in development of newer anti-trypanosomal drug with reduced toxicity, improved efficacy and pharmacokinetic profile. As the drug discovery and development process is expensive in terms of time and money, the cross application of existing series of compounds with selective trypanocidal activity may be the best prospect to new anti-trypanosomal drugs in the short term. More emphasis has to be put in the field of CADD approaches for development of anti-trypanosomal agents as CADD study reduces the time and cost required for development of newer analogues.
